# Progress in the Practice of Medicine

**Published:** 1874-07

**Authors:** Norman Bridge

**Affiliations:** Lecturer on Principles and Practice of Medicine, in Rush Medical College


ARTICLE II. — *Progress in the Practice of Medicine.* By NORMAN BRIDGE, M.D., Lecturer on Principles and Practice of Medicine, in Rush Medical College.

1. Croton-Chloral Hydrate.

(*a*) Its Action and Uses. By Dr. OSCAR LIEBREICH. (*British Medical Journal.*

(*b*) Its Properties and Uses. By Dr. J. BURNEY YEO. (*Lancet.*)

2. The Influence of Close Confinement in Prisons on the Production of Phthisis. By A. L. LEACH, M.D. (*Am. Jour. Med. Sciences*, April, 1874.).

3. Report of the Vaccine Department of the New York Dispensary for 1873. By FRANK P. FOSTER, M.D.

4. On Small Pox and Vaccination Experiences in the Franco-Prussian War. By MARTIN LUTHER, M.D., Reading, Pa. (*Med. and Surg. Reporter.*

5. Report on the Yellow Fever Epidemic of 1873, at Shreveport, La. By Drs. SNELL, PENNER and DAVIS, a Committee appointed for the purpose by the local Medical Society.

6. The Distribution and Natural History of Yellow Fever in the United States; with Chart showing elevations of localities where it has appeared from 1668 to 1874. By J. M. TONER, M.D. (*Annual Report Sup. Surgeon U. S. Marine Hospitals.*)

7. A Case of Excessive Eructation. By A. L. LEACH, M.D. (*Medical Times*, April 18.)

8. A Speculation on the Operative Treatment of Non-Traumatic Peritonitis. By WM. O'GORMAN, M.D. (*Transactions of the Medical Society of New Jersey, for* 1873.)

9. Hay Fever Successfully Treated. By T. C. HOOVER, M.D. (*Am. Jour. Med. Sciences*, April, 1874.)

10. Biliary Concretions Dissolved. By E. BURD, M.D. (*Med. and Surg. Reporter.*)

1. This comparatively new drug—the composition of which is formulated as $C_4\ H_3\ Cl_3\ O$, it being a chlorated aldehyde of crotonic acid ($C_4\ H_6\ O_2$)—has been the subject of experiments by Dr. Liebreich. Given to maniacs during an attack of mania, the patients sat quietly in their chairs sleeping during two hours. In tic-douloureux it leads to the cessation of pain before sleep is induced—differing in this from hydrate of chloral. The indications for its use are in cases where hydrate of chloral is inapplicable on account of heart disease, in cases of neuralgia of the nervus trigeminus and in cases where very large doses of chloral are necessary to produce sleep.

The drug, he thinks, acts by being decomposed first into a trichlorated body (allyl-chloroform) and then rapidly into a bichlorated body (bichlorallylene) in which condition it produces its effect on the nervous centre. Trichlorated substances and chloroform act first on the brain, then on the spinal cord, and finally on the heart—in this way acts hydrate of chloral. Bichlorated substances, on the other hand, act on the brain, spinal cord and medulla oblongata, but not on the heart—in this way acts croton-chloral; and this explains how a medicinal dose does not affect either circulation or respiration.

Dr. Yeo has made extensive observations on the effect of the croton-chloral. It is a "remedy of remarkable efficacy in some cases of neuralgia of the branches of the nervus trigeminus," says he, and it may afford relief in other forms of obstinate neuralgia. It has little effect in purely rheumatic cases, and is of no use in cases known as hysterical. For irritating cough, such as the night cough of phthisis, it is valuable. As an hypnotic it is variable in its effect, requiring a variable dose—one to ten grains—and being less reliable than the hydrate of chloral. The dose is to be regulated by the effect produced, the limit being ten grains. In severe neuralgias five grains may be given every half hour.

2. Dr. Leach has compiled statistics bearing on this subject from ten different prisons in this country. Nearly 46 per cent. of deaths in these institutions are from consumption. Many convicts are yearly pardoned because they have this disease, and many on being discharged go home to die of it; so that 46 per cent. is really below the actual rate of mortality from phthisis.

In some States the pardoning of phthisical convicts is common, in others it is not; and in the latter, the mortality in the prisons is greater.

In closely crowded cities in America, the deaths from phthisis do not exceed 15 per cent.—less than one-third that in prisons.

The physician at the Sing Sing prison reports that fully half those dying of phthisis under his care "have been in prison from one to five times before." But Dr. L. is doubtless correct that, "after allowing for the dissipation and abuse of the lives of criminals as a cause, still we are forced to the conviction that prison life is prolific in producing phthisis."

3. Dr. Foster finds the dried lymph vastly better and more sure of taking than dried crusts or capillary tubes. With this vaccine the success in 824 primary vaccinations was 87.69 per cent., and in 1,785 secondary vaccinations, 70.76 per cent. Much of the lymph used was two or three weeks old, the result with such being nearly as good as with that only as many days old.

4. The writer of this paper examined 536 cases of variolous disease in hospitals in the field; 401 had been vaccinated, 25 had never been vaccinated. Of those vaccinated, 3.75 per cent. died; of those re-vaccinated, 1.75 per cent. died, while of those unvaccinated the deaths were 68 per cent.

On computations made from Dr. L.'s figures, it is found that of the cases set down as having *good cicatrices*, 0.91 per cent. died, while of those having poor cicatrices, 17.56 per cent. died. The emphasis of figures cannot be increased.

5. The report of these Shreveport physicians shows, to their satisfaction, that the disease was brought directly from Havana on shipboard by way of New Orleans. The committee trace it by a direct chain of communication from patient to patient until it reached Shreveport. They also state that the city had been for some time in a despicably bad sanitary condition. This was in some measure due to a failure of the system of drainage which had been attempted to be introduced.

The first case occurred Aug. 15th; by the 25th of August, cases were occurring rapidly; the disease became epidemic Sept. 1st, and was killed by frost Oct. 20th. So great was the stampede, that of 12,000 inhabitants only 4,500 remained, and of these, 3,000 were attacked with the fever and 729 died. The whites were no more generally attacked than the negroes, but the fatality with them was five times as great. Men suffered more than women, and people from 20 to 35 years old suffered the greatest mortality. The deaths to those attacked were about 26 per cent. Death usually occurred in four or five days from the beginning of the attack. Of the 125 deaths, 85 are declared to have died with black vomit, and 19 with suppression of urine.

No treatment was found so good as *rest.* Mercury and quinine did injury rather than good.

6. Yellow fever in the United States has never appeared at an elevation of more than 700 feet above the sea level, the greater part of the territory visited by it has been less than 100 feet in elevation above this point.

7. Dr. L. reports a case of evident insolation in a laborer, 28 years old, in whom there was the most remarkable phenomenon of eructation of gas. The patient was attacked July 7th, at noon. In the evening he was better; next day resumed work; at 4 P. M. was seized again, had headache, vomiting and *belching of wind.* Next day he worked a little and the bad symptoms reappeared, which compelled him permanently to stop laboring. For a month he was eructing wind almost constantly. He would pass whole nights without sleep, when he could only be quieted with medicines.

At this time, Dr. L. found him "profoundly anæmic, with a cadaverous appearance." Respiration was difficult; gas was constantly passing from the stomach with a characteristic noise. Talking was interrupted by passages of gas, and he could not lie down for fear of choking, having slept in the sitting posture for some time. Everything he ate was eructed with the gas, and he had lost since his sun-stroke twenty-six pounds in weight. He was made worse by contact with the sun's rays. With the use of charcoal and tonics recovery was rapid.

Dr. L. is doubtless correct, that the brain was "the primary seat of trouble." The effect of disturbance of the brain in producing eruction is often remarkable. The reporter hereof has frequently seen in a member of his own family during an attack of sick-headache as abundant an eructation as that described by Dr. L., and continuing for an hour or more without interruption; and this, too, at times when the stomach had not for many hours received any food.

8. In this paper, a new surgical procedure is suggested for the disease named: opening the abdominal cavity by an incision for the escape of any effusion or other irritating matter. No cases of the operation are reported, but abundant facts and authorities are cited to show that the fatality of peritonitis is frequently due to the original irritation not being removed or increasing; and that the effusions due ordinarily to inflammation are usually irritants, especially if fluid and not readily reabsorbed.

In ovariotomy, peritonitis is no bar to operation. Wells has repeatedly operated under such circumstances, carefully removing all effusion, and 70 per cent. of his patients have recovered. Dr. Peaslee's experience with washing out the abdominal cavity with bland fluids and removing all irritating substances has been favorable, suggesting the impunity with which the peritoneum may be opened, provided irritating things be removed. Certain it is that, if in cases of non-traumatic peritonitis, as great a per cent. of recoveries could be insured by the operation advised as Wells has saved of his patients who had peritonitis at the time of operation, it would show a less rate of mortality than is now the experience.

9. Dr. H. reports four cases of cure of hay fever by atomized inhalations. One was a case of eleven years' standing ; the attacks occurring on the 18th of August annually and continuing six weeks. The solution used was, ten grs. chlorate of potassa and two grs. sulphate of morphia to the ounce of water. The relief was instantaneous ; the measure was frequently repeated for five days, when it was alternated with one of a solution of bromide of potassium (30 grs. to the ounce). In twenty days the case was well.

Another case had continued six years. Only the morphine solution was used, and the cure was complete. Two other cases were similarly treated and with like success.

10. A case is here reported of a woman 44 years old, who for twenty years had had colic, due to passage of gall-stones one or more times each week. She suffered greatly, was much debilitated, and had received many kinds of treatment.

In the intervals of the colic, Dr. B. prescribed equal parts Venice turpentine and ether, a teaspoonful being taken every morning fasting. Under this treatment, the attacks became less frequent, finally ceased, and the woman grew fleshy and became pregnant. She discontinued the medicine and in fifteen months (January, 1873) had another attack, since which time she has taken the drugs and has been free from colic. Dr. B. thinks the agents used dissolved the gall-stones.

This appears like a genuine case, in addition to those heretofore reported, of the cessation of this painful disease under the protracted use of the ethers, chloroform and the terebinthinate preparations.

